# Malaria *v.* More Recognisable Causes of Disease

**Published:** 1889-12-21

**Authors:** 


### LONDON EPIDEMIOLOGICAL SOCIETY.

### MALARIA, *VERSUS* MORE RECOGNISABLE CAUSES OF DISEASE.

Abstract of an address delivered by Sir William Moore, K.C.I.E., on December 11, 1889, Sir Thomas Crawford, K.C.B., in the chair.

Sir William Moore commenced his address by remarking on the number of maladies which had been attributed to malaria, from the most severe, such as cholera and sunstroke, to trivial affections, such as slight intermittents and evanescent neuralgias. But malaria had not yet been demonstrated to exist as an entity. It had been regarded as a vapour, as sulphuretted hydrogen, as carbonic oxide, etc., and, lastly, as a bacillus. But Sir William Moore could not regard the bacillus malariæ of the Italian investigators as the true germ, any more than he was prepared to admit the assertions of Salisbury, made years back, that malaria was a spore of a species of palmella. If the bacillus malariæ of Klebs and Crudeli were accepted as the malaria germ it entailed the belief that all kinds of soils were capable of producing it. For the semi-desert districts of Western India, where water is two or three hundred feet from the surface, and where there is scarcely any vegetation, and where there is no underlying stratum of water-bearing soil, are as prolific of fever as the Koncan, where water is three or four feet from the surface, where vegetation is most profuse, and where the soil is alluvial. Moreover, malarious fevers prevail on rocky sites, even on granite rock. The bacillus malariæ had not been found everywhere where malarious fevers prevail, and, notwithstanding all that had been advanced on the subject, it had not been proved that inoculation of the bacillus malariæ would induce so-called malarious fever in the human subject. It had been stated the malarious fever might result in half an hour after exposure to malaria. But the bacillus malariæ required time to germinate from their reputed homes in the spleen and marrow of the bones. Because a peculiar appearance was found in the blood, this was no reason for stating it was the cause of a disease. Ten per cent. of the Chinese have filaria in the blood, but do not suffer from elephantiasis, which filaria has been supposed to cause. Malarious fevers always mostly prevailed at the season when the greatest changes of temperature occurred, or at the time when a fall of temperature happened after a period of continued heat. And more especially so if damp were associated with fall of temperature. This held good not only in the tropics, but in semi-tropical, and in those temperate climes where malarious diseases had been noted. The cutaneous surface of Europeans, especially in hot climates, was rendered so sensitive by continued heat, and so debilitated by excessive action, that persons were affected by the slightest fall of temperature. And, moreover, the clothing worn in the East did not sufficiently protect from falls of temperature. There were very few cases of fever prior to which some exposure to damp and cold could not be traced. The result would be the same, whether there is such an entity as malaria or whether there is not. Referring to the somewhat questionable immunity of certain natives of tropical climates from malarious fevers, it was remarked that natives of India do not enjoy any such freedom, while negroes who have been asserted to enjoy such freedom possess a peculiarly unsensitive surface, excreting a large amount of sebaceous matter, and also they increase this unsensitive condition of the skin by anointing themselves with greasy substances. With regard to periodicity being a characteristic of malarious disease it was remarked that periodicity pertains to all nature, and that attacks of so-called malarious disease—even of intermittent fever—were so irregular as to lose all claim to periodicity, which implies regularity. If epidemics of so-called malarious fever among troops were referred to, it would be found that there were other causes of disease in operation. Such were barracks apparently designed so as to expose the men to the greatest continued heat and the greatest degree of cold, exposure to the night air, perhaps without tents, unsuitable clothing, getting wet from rain, often insufficient food and fatigue. These and others which might be named are recognisable causes of disease, and they would excite disease whether malaria were present or not. Moreover, after exposure to a chill, one man would suffer from an ordinary cold, another from an intermittent, a third from dysentery, a fourth from rheumatism, and a fifth from liver. If malaria were the cause, all should suffer from intermittent, for the other maladies named were not generally attributed to malaria, although, in common with almost every malady in the nomenclature, they had been so attributed. The good effected by drainage was not the diminution of malaria, but the lessening of damp, cold, and liability to chill, conditions which would excite disease whether mal[illegible]ria were present or not. It was the same when malarious fever prevailed extensively among civil populations. And there were few individual cases of fevers which could not be traced to a chill. The more a temperate climate approached the characteristics of the tropics the more the so-called malarial diseases. Hence in those temperate climates in which it had occurred it did so in the autumnal season, when hot days were succeeded by cold, damp nights. But the intensity of malarial fever declines as we proceed either north or south of the equator. Degrees of malarious fever, from the agues of Lincolnshire to the remittents of the tropics, may be estimated by the degree of solar heat, which implies vicissitudes between the night and day temperature, and the principal feature distinguishing so-called malarious places from healthy places was humidity. Sir W. Moore felt his inability to distinguish malarial from climatic influences. It had taken him some time to unlearn what had been taught in his youth. But if philosophers had been content to answer problems by reference to a hypothetical agency, our knowledge of many things would have been much less than it is. His position was briefly this: that so-called malarious fevers are caused by sudden abstraction of heat or chill, under the influence of cold, and more especially damp cold; that these effects of chill are most marked in hot climates, because of the antecedent exposure to great solar heat, the anæmia and skin debility resulting from heat, and the disregard of suitable precautions; that the individual malady will be simply of a febrile character in the absence of diseased predisposed conditions of any internal organ; that the febrile paroxysm will be repeated or not, according as temperament, heredity, habits, mental condition, anæmia, recurring chill, one or more favour such repetition; that so-called malarious cachexia, or anæmia, is caused by continued heat and not by malaria. In the discussion which ensued, Sir Thomas Crawford, Surgeon-General Lawson, Deputy Surgeon-General Scriven, and several others took part.

